# Intrinsic fluctuations of reinforcement learning promote cooperation

**DOI:** 10.1038/s41598-023-27672-7

**Published:** 2023-01-24

**Authors:** Wolfram Barfuss, Janusz M. Meylahn

**Affiliations:** 1 Tübingen AI Center, University of Tübingen, Tübingen, Germany; 2 Department of Applied Mathematics, University of Twente, Enschede, The Netherlands; 3 Dutch Institute of Emergent Phenomena, University of Amsterdam, Amsterdam, The Netherlands

**Keywords:** Evolutionary theory, Applied mathematics, Computer science, Statistical physics, thermodynamics and nonlinear dynamics

## Abstract

In this work, we ask for and answer what makes classical temporal-difference reinforcement learning with $\epsilon$-greedy strategies cooperative. Cooperating in social dilemma situations is vital for animals, humans, and machines. While evolutionary theory revealed a range of mechanisms promoting cooperation, the conditions under which agents learn to cooperate are contested. Here, we demonstrate which and how individual elements of the multi-agent learning setting lead to cooperation. We use the iterated Prisoner’s dilemma with one-period memory as a testbed. Each of the two learning agents learns a strategy that conditions the following action choices on both agents’ action choices of the last round. We find that next to a high caring for future rewards, a low exploration rate, and a small learning rate, it is primarily intrinsic stochastic fluctuations of the reinforcement learning process which double the final rate of cooperation to up to 80%. Thus, inherent noise is not a necessary evil of the iterative learning process. It is a critical asset for the learning of cooperation. However, we also point out the trade-off between a high likelihood of cooperative behavior and achieving this in a reasonable amount of time. Our findings are relevant for purposefully designing cooperative algorithms and regulating undesired collusive effects.

## Introduction

Problems of cooperation are ubiquitous and essential, for biological phenomena, as in the evolution of cooperation under natural selection, for human behavior, such as in cartel pricing or traffic, and increasingly so for intelligent machines with automated trading and self-driving cars^1–3^. In social dilemmas, individual incentives and collective welfare are not aligned. Individuals profit from exploiting others or fear being exploited by others, while at the same time, the collective welfare is maximized if all choose to cooperate^4^.

Understanding the conditions under which self-learning agents learn to cooperate spontaneously—without explicit intent to do so—is critical for three reasons: (1) It provides an alternative route to the emergence of (human and animal) cooperation when an evolutionary explanation is unlikely. (2) It guides the design of intelligent self-learning algorithms, which are supposed to be cooperative. (3) It provides policymakers and regulators the necessary background to design novel anti-trust legislation against undesirable collusion, e.g., in algorithmic pricing situations, where not doing so could lead to significant loss of consumer welfare^5^.

While evolutionary theory revealed a range of mechanisms that promote cooperative behavior, from direct and indirect reciprocity to spatial and network effects^6–10^, the conditions under which individually learning agents learn to cooperate are contested. Some works suggest that independent reinforcement learning agents are capable of spontaneously cooperating without explicit intent to do so^11–20^. Other works argue that the emergence of cooperation from independent learning agents is unlikely^21–25^, and therefore specific algorithmic features are required to promote cooperation^26–38^. As such, reinforcement learning variants called *aspiration learning*, which go back to a seminal work in psychology from Bush and Mosteller^39^, have been extensively investigated in social dilemmas. Whether two co-players learn to cooperate depends on the (dynamics of the) aspiration level^40–42^. This finding has been confirmed and extended to spatial or networked social dilemmas^43–46^. Aspiration learning has also been found to explain human play in behavioral experiments well^13,14^. However, comparably little is known about whether, when, and how cooperative behavior spontaneously emerges from the reinforcement learning variants called *temporal-difference learning*, which are extensively used in machine learning applications and is the dominant model used to explain neuroscientific experiments^47^.

**Figure 1:**
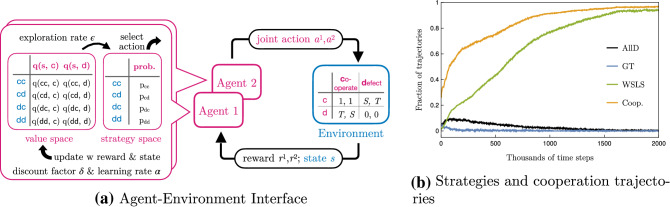
Overview. (**a**) Model sketch. (**b**) Fraction of 1000 samples from random initial state-action values in each of the three equilibria (All-Defect (AllD) in black, Grim-Trigger (GT) in blue, and Win-Stay, Lose-Shift (WSLS) in green) as a function of time when using $\epsilon$-greedy temporal-difference learning with $\epsilon =0.01, \delta =0.98$ $\alpha =0.1$. The fraction of times that both Q-learners cooperated in the last thousand time periods averaged over 1000 sample trajectories in light gray. Environment parameters are $T=1.5$ and $S=-0.2$.

In this work, we ask when and how cooperative behavior spontaneously emerges from temporal-difference learning with $\epsilon$-greedy strategy functions. This question is motivated by recent work on algorithmic collusion^48^ and the fact that $\epsilon$-greedy strategies are frequently used in machine learning^49^. The problem is that reinforcement learning is typically highly stochastic and data-inefficient, making it challenging to understand which features are decisive for learning cooperation. We solve this problem by dissecting the reinforcement learning processes into three parts using multiple mathematical techniques.

First, we consider the *stability* of strategies under reinforcement learning. We analytically derive when strategies are stable given how much the agents care for future rewards and explore the environment. Only one out of the three possible, stable strategies supports cooperation robustly.

Second, we consider the *learnability* of this equilibrium. We use deterministic strategy-average learning dynamics to compute the size of the basins-of-attraction given the agents’ learning rate. We find a maximum of approx. 40–50% of robust cooperation.

Third and last, we consider the *stochasticity* of the learning process. We simulate a stochastic batch-learning algorithm and find that the cooperative equilibrium steadily increases to 80-100%. Thus, a significant fraction of trials reaching the cooperative equilibrium must be due to the inherent fluctuations of the reinforcement learning process.

### Learning algorithm and environment

We consider the generalized and advantageous temporal-difference reinforcement learning algorithm *Expected SARSA*^49–51^ with $\epsilon$-greedy exploration. At each time step *t*, agent $i \in \{1,2\}$ chooses between two possible actions, $a \in \mathcal {A}^1 = \mathcal {A}^2 = \{\textsf {c}, \textsf {d}\}$, which represent a cooperative or a defective act. Given the joint action $\varvec{a} = \{a^1, a^2\}$ and the current state of the environment $s \in \mathcal S$, each agent receives a payoff or reward $r^i(s, \varvec{a})$ and the environment transitions to a new state $s' \in \mathcal S$ with probability $p(s'| \varvec{a}, s)$. Agent *i* chooses action *a* with frequency $x^i_t(a|s)$ which depends on the current environmental state $s \in \mathcal S$.

Agents derive these frequencies $x^i_t(a|s)$ from their state-action values $q^i_t(s, a)$ according to the $\epsilon$-greedy exploration scheme. Each agent selects the action with the largest state-action value with probability $1-\epsilon$, and with probability $\epsilon$, it selects an action uniformly at random. For the two-action case,

1 $$\begin{aligned} x^i_t(\textsf{c}|s) = {\left\{ \begin{array}{ll} 1-\epsilon /2 &{}\text {if }q^i_t(\textsf{c},a) > q^i_t(\textsf{d},a)\\ \epsilon /2 &{}\text {otherwise } \end{array}\right. }. \end{aligned}$$

$x^i_t(\textsf{d}|s)$ is defined analogously. The parameter $\epsilon$ regulates the exploration-exploitation trade-off.

The state-action values are updated after each time step as,

2 $$\begin{aligned} q^i_{t+1}(s_t, a_t) =&(1-\alpha )q^i_{t}(s_t, a_t) +\alpha \Big [r^i_t+\delta \sum _a x^i_t(a|s_{t+1}) q^i_t(s_{t+1}, a) \Big ], \end{aligned}$$

where the parameter $\alpha \in [0,1]$ denotes the learning rate, $r^i_t = r^i(s_t, \varvec{a}_t)$ denotes the rewards agent *i* receives at time step *t* and $\delta \in [0,1)$ denotes the agents’ discount factor, regulating how much they care for future rewards. For simplicity, we consider homogeneous and constant parameters $\alpha , \epsilon , \delta$ during the learning process.

The environment we study is the iterated Prisoner’s Dilemma. It is perhaps the most iconic and straightforward model system to investigate the preconditions for cooperative behavior, with an established body of research in fields as diverse as political science and evolutionary biology^52^. Because of its simplicity in carving out the tensions between individual incentives and collective welfare, we use it here as a model system to highlight an effect that is, therefore, likely to exist in other larger systems that retain similar tensions between individual incentives and collective welfare. Specifically, we use reward matrices given by, 
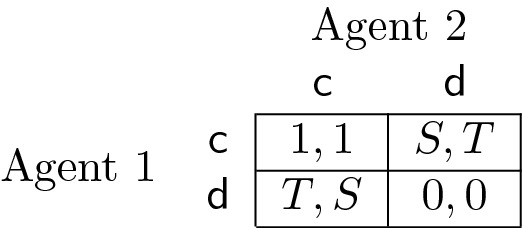


with $T>1>0>S$. The rewards for each combination of actions are written in the cells of the matrix. Each cell’s first (second) element denotes the payoff for agent 1 (2). With $T>1$ and $S<0$, each agent prefers defection over cooperation, regardless of what the other agent is doing. The dilemma is that both agents could achieve a higher reward if both cooperate.

However, when the game is repeated for multiple rounds, agents can condition their frequencies of choosing actions on the actions of past rounds, and mutual defection is no longer inevitable. A famous example is the Tit-for-Tat strategy^6^, in which you cooperate if your co-player cooperated, and you defect if your co-player defected in the last round.

We are interested in how two reinforcement learning agents endogenously learn such memory-1 strategies. Therefore, we embed the stateless Prisoner’s Dilemma game into an environment where the current environmental state $s_t=a^1_{t-1}a^2_{t-1}$ signals the actions of the last round. Thus, the state set reads $\mathcal S = \{\textsf{cc}, \textsf{cd}, \textsf{dc}, \textsf{dd}\}$. Fig. 1a illustrates our setting.

**Figure 2:**
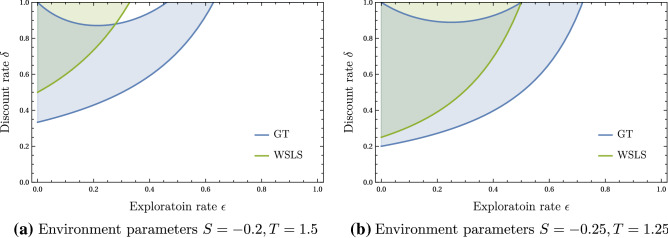
Stability parameter space. Phase diagrams show which strategy equilibrium solutions are possible. The All-Defect (AllD) solution is possible everywhere. The Grim-Trigger (GT) solution is possible in the blue region, and the Win-Stay, Lose-Shift (WSLS) strategy is possible in the green region.

Recent work has shown that only three strategy pairs are an equilibrium for $\epsilon$-greedy temporal-difference learning with one-period memory in the iterated Prisoner’s Dilemma in the small exploration rate limit^53,54^. Interestingly, the Tit-for-Tat strategy is not an equilibrium. The three strategies are All-Defect (AllD), Grim-Trigger (GT), and Win-Stay, Lose-Shift (WSLS). In AllD, both agents defect regardless of the previous period. In GT, agents play AllD except when both players cooperated in the last period, which is answered by cooperation. And in WSLS, agents play GT, except that cooperation also follows a previous period of both players defecting. Only the WSLS equilibrium leads to robust cooperation. Under the GT strategy, both agents keep cooperating, given that they have cooperated in the last round. But erroneous or exploration moves make it more likely to switch from full cooperation to full defection than switching from full defection back to full cooperation.

Figure 1b shows the running average of the fraction of times both players cooperated (yellow) as a function of time, beginning from 1000 random initial state-action values. The trajectories of the fraction of the three stable strategies are likewise shown. In the end, the agents cooperate almost always. This shows that temporal-difference reinforcement learning can spontaneously learn to cooperate. However, it leaves open the question of why the agents learn to cooperate and which features of the learning algorithm are decisive for its ability to cooperate. In particular, the effects of the exploration and learning rates, $\epsilon$ and $\alpha$, and the intrinsic stochasticity of the reinforcement learning process remain unclear.

## Dissecting reinforcement learning

To shed light on the questions raised above, we will dissect the reinforcement learning processes into three parts. First, we consider the *stability* of strategy pairs under reinforcement learning, considering agents’ discount factor $\delta$ and the exploration rate $\epsilon$. Second, we analyze the *learnability* of this equilibrium, taking into account the learning rate $\alpha$. Third and last, we consider the *stochasticity* of the learning process by introducing a batch-learning variant of our temporal-difference reinforcement algorithm with a batch size parameter *K*.

### Stability

This section shows how the exploration rate $\epsilon$ affects the stability landscape. We analytically derive when strategy pairs are stable under the reinforcement learning update outside the small exploration limit. To do so, we refine the mathematical technique of *Mutual Best-Response Networks*^54^. With this method, we construct a directed network where the nodes represent the strategy pairs, and the edges represent a best-response relationship (see "Methods").

We find that AllD is always a solution. The condition for having WSLS as a solution is

3 $$\begin{aligned} \delta > \frac{2(T-1) + \epsilon (1-S-T)}{2(1 - \epsilon )^{2}}, \end{aligned}$$

while the condition for having GT as a solution is

4 $$\begin{aligned} \frac{2 S + \epsilon (1-S-T)}{(1 - \epsilon ) [(2-\epsilon )S -\epsilon T]}> \delta > \frac{2(T-1) + \epsilon (1-S-T)}{(1-\epsilon )(2T -\epsilon [S+T])}. \end{aligned}$$

The condition for WSLS (Eq. 3) is always greater than the lower bound condition for GT (Eq. 4). This means the robustly cooperative WSLS always requires a higher discount factor than the GT strategy equilibrium.

Figure 2 illustrates when the three equilibrium strategy pairs, AllD, GT, and WSLS, are stable, given the discount factor $\delta$ and the exploration rate $\epsilon$. The cooperative WSLS strategy pair is stable when $\delta$ is high, and $\epsilon$ is small. The GT strategy pair also requires a high $\delta$ and a small $\epsilon$ to become stable, yet, with less extreme parameter values. Interestingly, for large discount factors $\delta$, our theory predicts the GT equilibrium to lose stability for exploration rates $\epsilon$ between 0.0 and around 0.4 for the values chosen for *T* and *S* in Fig. 2. The AllD equilibrium is always stable.

### Learnability

In this section, we show how the learning rate $\alpha$ affects the learnability of the robustly cooperative WSLS equilibrium. With learnability, we mean the likelihood that the learning process reaches an equilibrium, i.e., the size of the state-action-value space from which the WSLS is learned. Following the edges along the *Mutual Best-Response Networks* represent the deterministic dynamics of a reinforcement learning algorithm, learning with perfect information and a learning rate of $\alpha =1$^54^. The maximum learnability of the WSLS equilibrium under these dynamics, as given by its basin of attraction, over all possible parameters ($T, S, \epsilon$ and $\delta$) is 0.015625 (see "Methods"). Cooperation is thus not very likely in this case.

**Figure 3:**
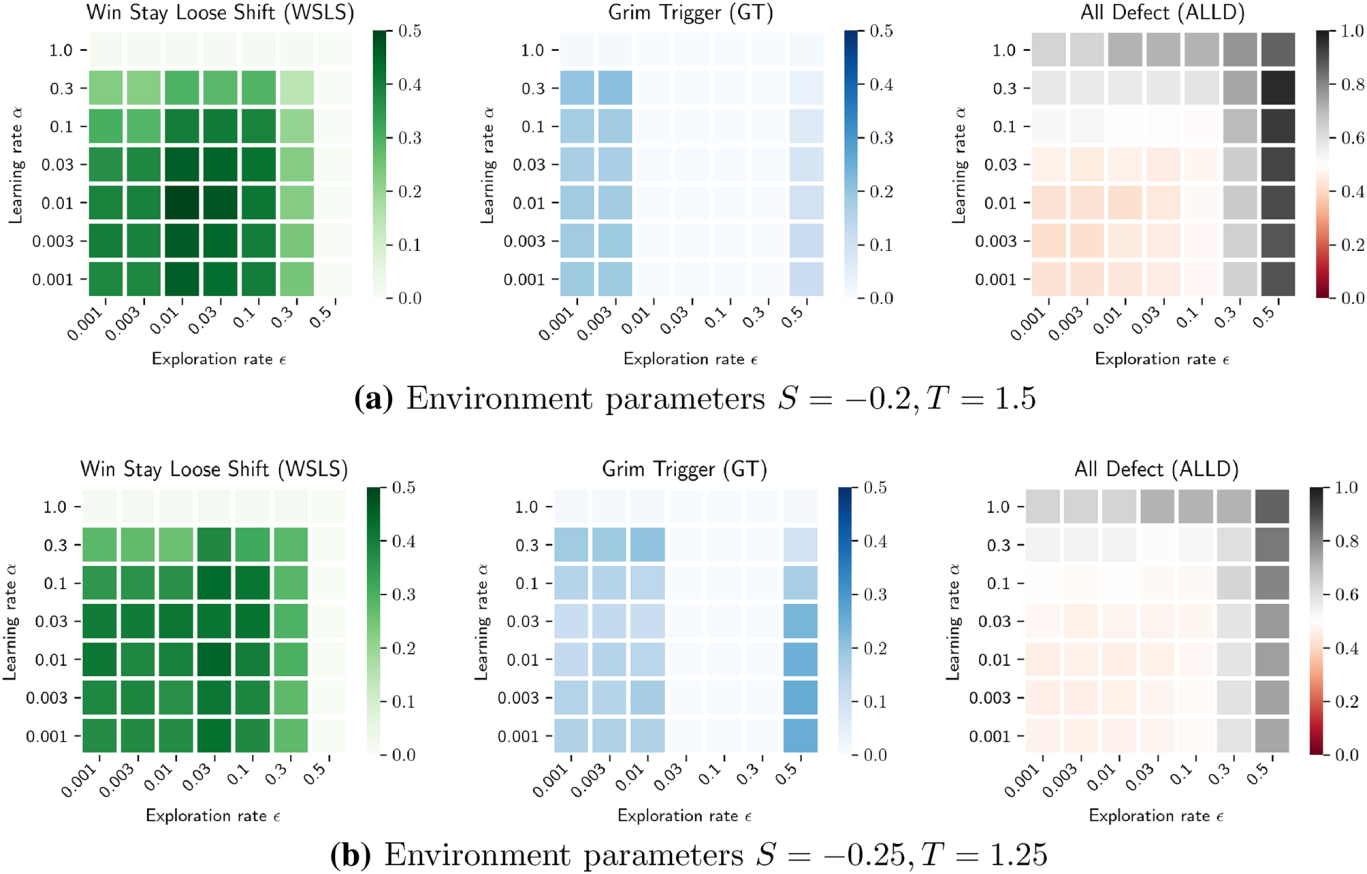
Learnability parameter spaces. Colors indicate which fraction of 250 random initial state-action values converges to the respective equilibria (the robustly cooperative WSLS in green on the left, GT in blue in the center, and AllD on the right) for a distinct parameter combination. Each plot portrays the parameter space spanned by the learning rate $\alpha$ versus the exploration rate $\epsilon$. The discount factor $\delta =0.99$.

To investigate the learning dynamics for a learning rate of $\alpha <1$, we refine the mathematical technique of deterministic reinforcement learning dynamics^55–57^. This method considers the mean-field of an infinite memory batch to construct idealized learning updates precisely in the direction of the strategy-average temporal-difference error. Previous work investigated the dynamics in strategy space. We formulate the dynamics in state-action-value space to account for $\epsilon$-greedy policies (see "Methods").

To estimate the size of the state-action-value space from which the agents learn WSLS, we let them start from 250 random initial state-action values (Fig. 3). Thus, with deterministic dynamics, the only randomness introduced in this section results from the initial state-action-value conditions.

Overall, we find that the robustly cooperative WSLS equilibrium is learned from a maximum of 40–50% of the state-action-value space, given the learning rate $\alpha$ and the exploration rate $\epsilon$ are not too large (Fig. 3, left plots in green). Values below 0.1 for each parameter are sufficient, independent of the environmental parameters investigated.

Furthermore, the deterministic learning dynamics confirm the non-trivial predictions of Fig. 2 that the GT strategy is unstable for intermediate values of the exploration rates $\epsilon$ and large discount factors $\delta$ (Fig. 3, center plots in blue). Fig. 3 also confirms the prediction that at exploration rates $\epsilon$ close to zero, the GT stability boundary is steeper for the environment with $S=-0.2$ and $T=1.5$ than for the environment with $S=-0.25$ and $T=1.25$ (Fig. 2). Grim Trigger is learned for exploration rate $\epsilon =0.01$ in the latter environment, but not in the former (Fig. 3).

Lastly, we find that outside the square of learning rate $\alpha =0.1$ and exploration rate $\epsilon =0.1$, more than half of the state-action-value space leads to the AllD equilibrium, independent of the environments investigated (Fig. 3, right plots). Inside this square, the AllD equilibrium no longer dominates the state-action-value space. Less than half of it leads to complete defection.

### Stochasticity

In this section, we show that intrinsic fluctuations of the typical online reinforcement learning process significantly improve the learnability of the robustly cooperative WSLS equilibrium. To be able to interpolate between fully online learning and deterministic learning, we refine the temporal-difference reinforcement learning algorithm (Eq. 2) with a memory batch of size $K \in \mathbb N$. Batch learning is a prominent algorithmic refinement because of its efficient use of collected data and the improved stability of the learning process when used with function approximation^58–60^. The agents store experiences (observed states, rewards, next states) of *K* time steps inside the memory batch and use their averages to get a more robust learning update of the state-action values (see "Methods"). The batch size *K* allows us to interpolate between the fully online learning algorithm (Eq. 2) for $K=1$ and the deterministic learning dynamics for $K=\infty$. We simulate the stochastic batch-learning algorithm for an exemplary set of parameters to showcase the effect intrinsic fluctuations can have on the learning of cooperation. Our goal here is not to optimize this set of parameters, as a thorough theoretical treatment of the resulting stochastic process is beyond the scope of this work.

**Figure 4:**
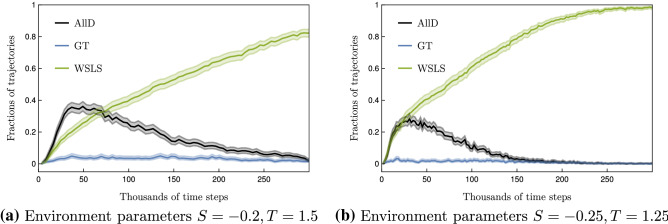
Stochasticity of batch learning. Fraction of 1000 from random initial state-action values in each of the three equilibria versus time steps for the sample-batch learning algorithm with $\epsilon =0.1$, $\delta =0.99, \alpha =0.3$ and $K=4096$ for (**a**) and $K=2048$ for (**b**). The shaded region shows a 95% confidence interval calculated using the Wilson Score^62^.

We find that intrinsic fluctuations significantly increase the level of the robustly cooperative WSLS equilibrium compared to the deterministic learning dynamics. At the same time, the batch learning agents require an order of magnitude fewer time steps to reach such high levels of cooperation than the batch-less online algorithm (Fig. 4). We observe that the fraction of the cooperative equilibrium steadily increases to over 80%, about twice the level reached with the deterministic learning dynamics in Fig 3. We hypothesize that the stability of the equilibria under noisy dynamics is a crucial factor. From Fig. 4, we see that the percentage of trajectories in the WSLS state increases. In contrast, for the two other strategies, the percentage first increases and then decreases (with occasional upward fluctuations). This suggests that the WSLS strategy pair is more stable than the other two, given this choice of environment and algorithm parameters.

Interestingly, Fig. 4 also shows that the high level of robust cooperation is reached on a time scale that is an order of magnitude shorter than that of the purely online algorithm (Eq. 2). Whereas the online algorithm takes in the order of $10^6$ time steps, the batch learning algorithm only requires the order of $10^5$ time steps to reach high cooperation levels. This is remarkable because, in the batch-learning simulation, we purposefully restrict the agents to update their strategies only after completing an entire batch. In practice, learning a strategy using a memory batch and learning a model of the environment will be more intertwined^61^, offering additional efficiency gains. This suggests the existence of a sweet spot between high levels of final cooperation and the time agents require to learn them.

To check the robustness of our results, we repeat the simulations of Fig. 4 for different combinations of the algorithm parameters $K, \alpha$ and $\epsilon$. The values we investigate are an increasing batch size $K\in \{1000, 2000, 3000, 4000, 5000, 6000, 7000, 8000, 9000, 10000\}$, for learning and exploration rate around the critical values 0.1 which are decisive for high levels of robust cooperation in the deterministic approximation (Fig. 3): learning rate $\alpha \in \{0.003, 0.006, 0.1, 0.2, 0.3\}$ and exploration rate $\epsilon \in \{0.003, 0.006, 0.1, 0.2, 0.3\}$. We record the fraction of trajectories (based on 1000 samples) in the WSLS strategy pair at time $2\times 10^{6}$. To get an indication of the speed at which the WSLS strategy pair is learned, we record the time at which the fraction of trajectories for a given set of parameters reaches 0.4.

**Figure 5:**
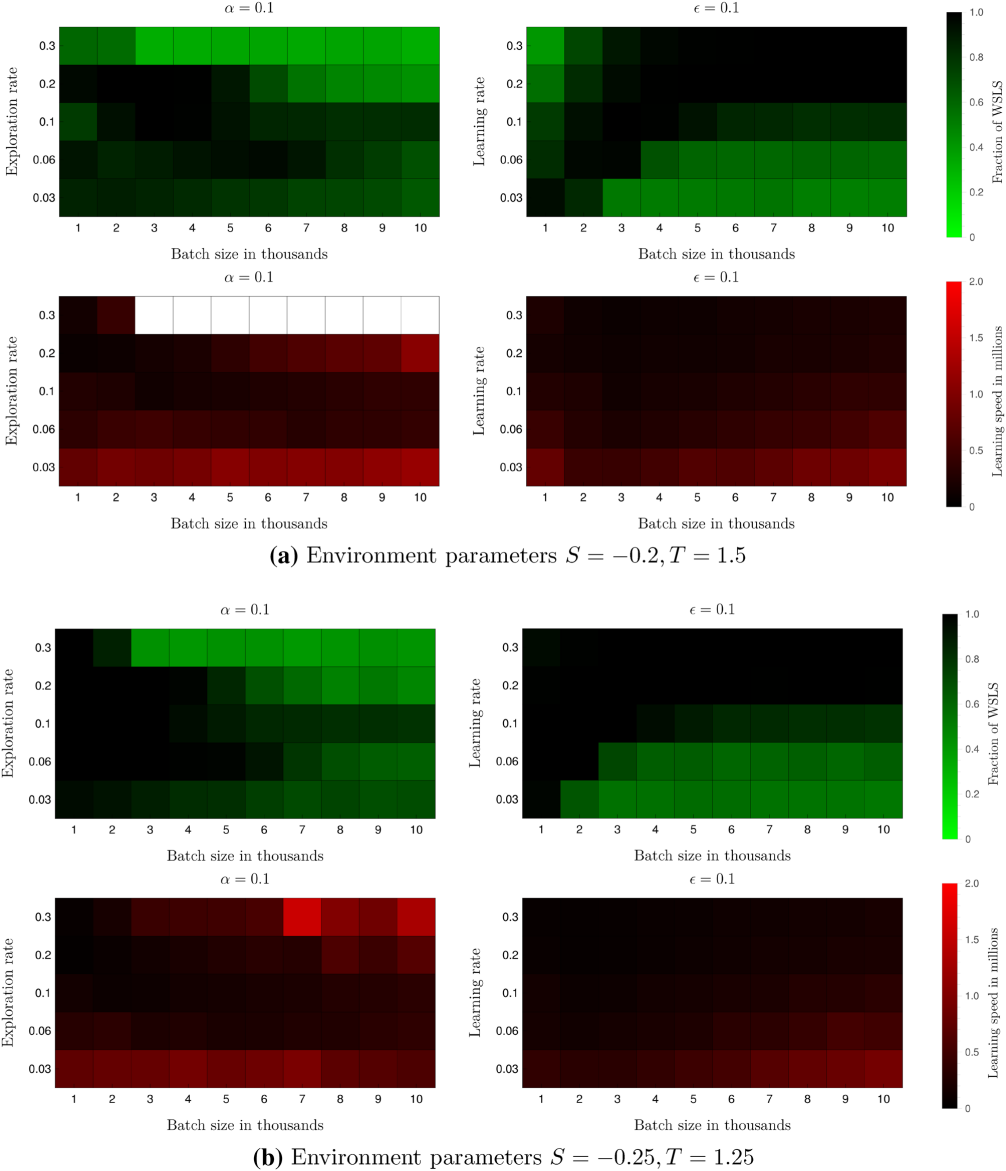
Robustness analysis for stochastic learning. The green plots show the fraction of trajectories (1000 samples) that end in the WSLS strategy pair at time $2\times 10^{6}$, and the red plots show the time it takes for the fraction of trajectories in the WSLS strategy pair to reach 0.4 in millions of time steps (we use white to represent trajectories that never reached 0.4). The x-axis always represents the batch size in thousands, and the y-axis represents either the learning rate $\alpha$ or the exploration rate $\epsilon$. In all cases, we set $\delta =0.99$.

In Fig. 5, we show the results for both environments around the critical parameter space point $(\epsilon , \alpha )=(0.1, 0.1)$. The rest of the results are presented in the *Supplementary Information*. We find that the fraction of trajectories in the WSLS strategy pair reaches values close to one for a large proportion of the investigated parameters. The results are thus robust to changes in the algorithm parameters.

Our robustness analysis also shows that intrinsic fluctuations do not make the other parameters irrelevant. We are able to draw some elementary conclusions regarding the combinations of parameters that lead to high levels of cooperation: (1) agents must not explore too much. Using an $\epsilon = 0.3$ leads to low levels of cooperation. (2) agents must not explore too little. We see that an $\epsilon = 0.03$ consistently leads to slower learning speeds than using intermediate values of the exploration rate. (3) larger learning rates lead to quicker learning speeds in the range of values we tested. In some cases, however, increasing the learning rate leads to lower levels of cooperation. The effect of changes in the batch size does not reveal a consistent pattern across the parameter ranges we tested. But if we restrict ourselves to the parameter values for the learning and exploration rates suggested by points (1)–(3), for example, $\alpha , \epsilon \in \{0.1, 0.2\}$, we see that an intermediate batch size of $K\in \{3000, 4000, 5000\}$ gives high levels of cooperation and achieves these quickly. Clearly, the interaction between these three parameters in how they influence the level of cooperation and the learning speed is complex. We leave a more detailed (theoretical) analysis of this interaction for future work.

## Discussion

### Contributions

In this article, we have shown that learning with imperfect, inherently noisy information is critical for the emergence of cooperation. We have done so by dissecting the widely used temporal-difference reinforcement learning process into three components.

First, cooperation can only be learned if a *stable* equilibrium supports it. We have shown how the existence of all possible equilibria depends on the combination of environmental parameters, *T*, *S*, the agents’ exploration rate, $\epsilon$, and how much they care for future rewards, $\delta$; under the assumption that the reinforcement learning update takes into account perfect information about the environment and the other agent’s current strategy. The robustly cooperative Win-Stay, Lose-Shift (WSLS) equilibrium requires a small exploration rate, $\epsilon$, and a large discount factor $\delta$ to be stable (Fig. 2), but it is not the only stable equilibrium.

Second, cooperation will only be *learned* if the WSLS equilibrium gets selected. This is more likely, the greater the size of the region of attraction leading to the WSLS equilibrium under the learning process. We have shown for a large discount factor $\delta$ how the likelihood of learning all possible equilibria depends on the combination of the agents’ learning and exploration rates $\alpha$ and $\epsilon$; as well as under the assumption that the reinforcement learning update takes into account perfect information about the environment and the other agent’s current strategy. The robustly cooperative Win-Stay, Lose-Shift (WSLS) equilibrium requires a small $\alpha <0.1$ and a small $\epsilon <0.1$ to achieve cooperation levels of 40–50% (Fig. 3). It is already interesting to observe that even though we give the reinforcement learners perfect information about the environment and the other agent’s strategy, not using all of it for a learning update ($\alpha < 1$) is required to achieve cooperation levels of 40–50%.

Third, we have shown that the internal *stochasticity* of the learning process significantly improves the learnability of the robustly cooperative WSLS equilibrium. We have done so by simulating a sample batch version of the algorithm. Surprisingly, this algorithm learns to cooperate on a significantly shorter time scale than the online algorithm (Fig. 4). This highlights an essential trade-off between the cooperative learning outcome and the time it takes the agents to learn this outcome. For example, our finding suggests that in the seminal work of Sandholm and Crites^21^ the number of iterations and the amount of exploration for each trial was set too small to observe a high cooperation rate between two learning agents.

The fact that intrinsic fluctuations of reinforcement learning promote cooperation is remarkable if we consider learning as a necessary tool to approximate an optimal solution when we don’t have all information about the environment available. Indeed, temporal-difference learning will always converge to an optimal solution, given a decreasing learning rate and sufficient exploration^49^. However, this is only true in a single-agent environment. There, learning serves as a means to overcome a lack of information for optimal decision-making. More information could only improve learning and decision-making.

For multi-agent learning, the situation is radically different. We have shown that learning with imperfect information is not a necessary evil to overcome a lack of knowledge about the environment. Intrinsic fluctuations in the learning process are a crucial asset to learning collectively high-rewarding, cooperative solutions.

Methodologically, obtaining our result was possible by two complementary tools for studying strategy-average reinforcement learning dynamics in stylized games. We introduced mutual best-response networks for describing the dynamics in the strategy space and strategy-average learning dynamics for describing the learning in the value space. These methods are not tailored to investigate the iterated Prisoner’s Dilemma. They are likewise applicable to derive insights from many other possible learning environments.

### Related work

Our main result, that intrinsic fluctuations in temporal-difference reinforcement learning promote cooperation, is in general agreement with the result that noise in biological systems is not negligible^63^. With respect to evolutionary and learning dynamics, it is important to distinguish different noise concepts.

Firstly, there is noise arising from suboptimal decision-making. In evolutionary game theory, such noise models the irrational or erroneous decision of players when adopting a less promising or rejecting a more promising strategy of another player. Such noise can be beneficial for cooperation^10,64,65^. For individual learning, the analogous noise concept arises from the need to deviate from the currently optimal course of action to further explore the environment and improve the current strategy. Thus, it is not necessarily irrational or erroneous to do so, but required for an individual learner. Analogous to evolutionary dynamics^64^, this exploration parameter can cause bifurcations towards highly desirable equilibria^66^. In our setting, the exploration rate, $\epsilon$, regulates this exploration-exploitation trade-off, and we show analytically that a small $\epsilon$ is required for robust cooperation (Fig. 2).

Secondly, external noise affecting the payoffs or rewards the agents receive can enhance cooperation in evolutionary dynamics^67,68^. Similarly, it was recently shown that external Lévy noise promotes cooperation^69^ in reinforcement learning.

Thirdly, noise in the perceptions of agents can affect cooperation in learning and evolutionary dynamics^70^. For example, inaccurate observations can lead to better learning outcomes in faster learning time, the stabilization of an otherwise chaotic learning process, and the mitigation of social dilemmas^71^ (Fig. SI 1).

Fourthly, there is the intrinsic noise of the evolutionary or learning process itself. In evolutionary game theory, such intrinsic noise arises because of finite populations, which can be highly beneficial for the evolution of cooperation^72^. With respect to learning dynamics, such intrinsic noise has been found to lead to noise-sustained cycling between cooperation and defection^73–75^. This is the noise concept we are referring to when we speak about intrinsic fluctuations, and we have shown empirically how these fluctuations can be highly beneficial for the learning of cooperation.

### Limitations and future work

Our results show that understanding the effects of intrinsic fluctuations in reinforcement learning is crucial in multi-agent systems. A formal treatment of these fluctuations is currently lacking and is an important avenue for future work.

The time scale on which the agents learn cooperation in our simulation with the sample batch algorithm is an order of magnitude faster than the online algorithm. Tuning the sample-batch-algorithm parameters, refining the algorithm with techniques such as optimism and leniency^37,76^, and using more refined model-based variants^61^ may further improve the learning speed.

Our work has focused on $\epsilon$-greedy learning policies, which differ significantly from softmax exploration. Studying the learning dynamics under such policies will determine whether the results are a feature of exploration in general or are specific to $\epsilon$-greedy exploration.

The environment of the iterated prisoner’s dilemma is paradigmatic, but certainly not the only environment for studying cooperation. Our methods lend themselves to be applied in a variety of settings, such as a pricing duopoly with a discrete price space^48^, public goods games or common-pool resource harvesting with more than two learning agents^77,78^, and social dilemma situations with changing external environments^79,80^.

### Practical implications

Our results highlight that both designers of cooperative algorithms and regulators of algorithmic collusion must not focus solely on the learning outcome, but also on the learning efficiency. The existence of (online) algorithms that learn to cooperate under self-play is not sufficient for them to be applied in practice unless cooperation occurs on reasonable time scales, and they can learn reasonable strategies against a large class of algorithms currently employed in practice^81^.

Overall, when designing sample batch algorithms, cooperation can be optimized, given the environment (*T* and *S*), by choosing $\delta$, $\epsilon$, $\alpha$, and *K* following three guiding criteria: (1) the cooperative equilibrium exists and has a relatively large basin of attraction, (2) the difference in stability between the cooperative equilibrium and the other equilibria is maximized in favor of the cooperative equilibrium, and that (3) the time scale on which cooperation is achieved is minimized.

## Methods

### Mutual best-response networks (MBRN)

An $\epsilon$-greedy strategy can be characterized by a pure strategy, determined using the ordering of state-action values, together with exploration. If $\epsilon$ is fixed, all possible $\epsilon$-greedy strategies can be enumerated and represented using a four-dimensional vector. Given that the opponent plays a fixed $\epsilon$-greedy strategy, we can solve the Bellman equations to obtain the $\epsilon$-greedy strategy that is a best response.

The state the system is in at any time, given two agents using an $\epsilon$-greedy strategy, is similarly characterized by an eight-dimensional vector representing both strategies. We refer to this eight-dimensional vector as a *strategy pair*. A mutual best-response to a strategy pair is a strategy pair in which both agents play a strategy that is an $\epsilon$-greedy best response to the opponent’s previous strategy. In this way, we construct a directed network (of 256 strategy pairs) with edges representing mutual best responses.

By considering all possible strategy pairs, we can tabulate which edges are possible in the resulting MBRN, as well as the conditions for their existence. Taking the intersection of all possible combinations of edge conditions splits the parameter space into regions, so each region corresponds to a different MBRN (similar to the phase diagrams in^54^). As a result, we can calculate maxima and minima over the entire parameter space by considering all possible MBRNs.

The structure of this network depends on the reward parameters (*T* and *S*), $\epsilon$, and $\delta$. Strategy pairs with self-loops are an equilibrium under mutual best-response dynamics. By solving the Bellman equations self-consistently, we can determine the critical conditions at which strategy pairs become equilibria.

We define the fraction of strategy pairs that lead to an equilibrium under the mutual best-response dynamics as its basin of attraction. Given an initialization that selects an initial strategy pair uniformly at random from all possible strategy pairs, the basin of attraction of an equilibrium strategy pair represents the probability of ending in that strategy pair under the mutual best-response dynamics.

### Learning dynamics

In essence, deterministic temporal-difference learning dynamics use strategy averages instead of individual samples of obtained rewards and estimated next-state values. They model the idealized learning behavior of agents with an infinite memory batch^56^ or with separated time scales between the process of interaction and adaptation^57^. Existing learning dynamic equations with $\epsilon$-greedy strategies were derived only for stateless interactions^82^. State-full learning dynamics employed only softmax strategies^55^. In the following, we present the deterministic Expected SARSA equations for state-full environments with $\epsilon$-greedy strategies in discrete time.

These dynamics operate in the joint state-action-value space $\varvec{q} =\bigotimes _{i,s,a} q^{i}(s, a)$. In order to formulate the strategy-average update of $\varvec{q}$ we define the joint strategy $\varvec{x} =\bigotimes _{i,s,a} x^{i}(a|s)$ with $x^{i}(a|s)$ as the probability that agents *i* will take action *a* in state *s*. For $\epsilon$-greedy strategies, $\varvec{x}$ is uniquely determined by $\varvec{q}$ and $\epsilon$. To obtain deterministic dynamics, we need to derive the strategy-average version of the state-action update (Eq. 2),

5 $$\begin{aligned} q^{i}_{t+1}(s, a) = q^{i}_{t}(s, a)&+ \alpha \Big [r_{\varvec{x}_t}^{i}(a|s) +\delta \cdot {}^{\text {next}}q_{\varvec{x}_t}^{i}(s, a) - q^{i}_{t}(s, a)\Big ], \end{aligned}$$

where $r_{\varvec{x}_t}^{i}(s, a)$ is the strategy-average version of the current reward and ${}^{\text {next}}q_{\varvec{x}_t}^{i}(s, a)$ the strategy-average version of the expected value of the next state.

The strategy-average version of the current reward is obtained as

6 $$\begin{aligned} r_{\varvec{x}_t}^{i}(s, a) = \sum _{s'} \sum _{j \ne i} \prod _{a_j} x^{j}(a^j|s) \cdot p(s'|\varvec{a}, s) \cdot r^{i}(s'). \end{aligned}$$

For each agent *i*, taking action *a* in state *s* when all other agents *j* act according to their policies $x^{j}(a^j|s)$ causes the next state $s'$ via the *transition probability* $p(s'|\varvec{a}, s)$ at which agent *i* obtains the reward, $r^{i}(s')$.

Second, the strategy-average version of the expected value of the next state is likewise computed by averaging over all actions of the other agents and next states. For each agent *i* and state *s*, all other agents $j \ne i$ choose their action $a^j$ with probability $x^{j}(s,a^j)$. Consequently, the environment transitions to the next state $s'$ with probability $p(s'|\varvec{a}, s)$. At $s'$, the agent estimates the quality to be the average of $q_{\varvec{x}_t}^{i}(s',b)$ with respect to its own strategy. Mathematically, we write

7 $$\begin{aligned} {}^{\text {next}}q_{\varvec{x}_t}^{i}(s, a) :=&\sum _{a^j} \sum _{s'} \prod _{j \ne i} x^{j}( a^j|s) p(s'|\varvec{a}, s) \times \sum _{b}x^{i}(b|s') q_{\varvec{x}_t}^{i}(s', b). \end{aligned}$$

Here, we replace the quality estimates $q^{i}_{t}(s,a)$, which evolve in time *t* (Eq. 2), with the strategy-average state-action quality, $q_{\varvec{x}_t}^{i}(s, a)$, which is the expected discounted sum of future rewards from executing action *a* in state *s* and then following along the joint strategy $\varvec{x}$. It is obtained by adding the current strategy-average reward $r_{\varvec{x}_t}^{i}(s,a)$ to the discounted strategy-average state quality of the next state $v_{\varvec{x}_t}^{i}(s')$,

8 $$\begin{aligned} q_{\varvec{x}_t}^{i}(s,a) = r_{\varvec{x}_t}^{i}(s,a) + \ \delta \sum _{s'} p_{\varvec{x}_t}^{i}( s'| a^i, s) \cdot v_{\varvec{x}_t}^{i}(s'). \end{aligned}$$

Here, $p_{\varvec{x}_t}^{i}( s'| a^i, s)$ is agent *i*’s strategy-average transition probability to state $s'$ from state $s^i$ under action $a^i$. It is computed by averaging over all actions of the other agents. For each agent *i* at state *s*, selecting action $a^i$, all other agents $j \ne i$ select action $a^j$ with probability $x^{j}(a^j|s)$. Consequently, the environment will transition to the state $s'$ with probability $p(s'| \varvec{a}, s)$. Mathematically, we write

9 $$\begin{aligned} p_{\varvec{x}_t}^{i}( s'| a^i, s) = \sum _{a^j} \prod _{j \ne i} x^{j}(a^j|s) \cdot p(s'| \varvec{a}, s). \end{aligned}$$

Further, at Eq. (8), $v_{\varvec{x}_t}^{i}(s)$ is the strategy-average state quality, i.e., the expected discounted sum of future rewards from state *s* and then following along the joint strategy $\varvec{x}$. They are computed via matrix inversion according to

10 $$\begin{aligned} \varvec{v}_{\varvec{x}_t}^{i} = [ \varvec{\varvec{\mathbbm {1}}}_{|\mathcal {S}|} - \delta \varvec{p}_{\varvec{x}_t} ]^{-1} \varvec{r}_{\varvec{x}_t}^{i}, \end{aligned}$$

where $\varvec{v}_{\varvec{x}_t}^{i}$ denotes the $|\mathcal {S}|$-dimensional vector containing $v_{\varvec{x}_t}^{i}(s)$ in entry *s*, $\varvec{r}_{\varvec{x}_t}^{i}$ is defined analogously and $\varvec{p}_{\varvec{x}_t}$ is a $|\mathcal {S}|\times |\mathcal {S}|$ matrix containing $p_{\varvec{x}_t}(s, s')$ (defined in Eq. (11) below) at entry $(s, s')$. Eq. (10) is a direct conversion of the Bellman equation $v_{\varvec{x}_t}^{i}(s)= r_{\varvec{x}_t}^{i}(s) + \delta \sum _{s'} p_{\varvec{x}_t}(s, s') v_{\varvec{x}_t}^{i}(s')$, which expresses that the value of the current observation is the discount factor weighted average of the current payoff and the value of the next state. Bold symbols indicate that the corresponding object is a vector or matrix, and $\varvec{\varvec{\mathbbm {1}}}_Z$ is the *Z*-by-*Z* identity matrix.

The strategy-averaged transition matrix is denoted by $\varvec{p}_{\varvec{x}_t}$. The entry $p_{\varvec{x}_t}(s, s')$ indicates the probability that the environment will transition to state $s'$ after being in state *s*, given all agents follow the joint strategy $\varvec{x}$. We compute them by averaging over all actions from all agents,

11 $$\begin{aligned} p_{\varvec{x}_t}(s, s')= \sum _{a^j} \prod _{j} x^{j}(a|s) \cdot p(s'| \varvec{a}, s). \end{aligned}$$

Further, in Eq. (10), $r_{\varvec{x}_t}^{i}(s)$ denotes the strategy-average reward agent *i* obtains at state *s*. We compute them by averaging all actions from all agents and all next states. For each *i* at state *s*, all agents *j* choose action $a^j$ with probability $x^{j}(a^j|s)$. Hence, the environment transitions to the next state *s* with probability $p(s'|\varvec{a}, s)$ and agent *i* receives the reward $r(s')$,

12 $$\begin{aligned} r_{\varvec{x}_t}^{i}(s) := \sum _{a^j} \prod _{j} x^{j}(a^j|s) p(s'| \varvec{a}, s) r(s'). \end{aligned}$$

Note that the quality ${}^{\text {next}}q_{\varvec{x}_t}^{i}(s, a)$ depends on *s* and *a* although it is the strategy-averaged expected value of the next state.

We finally obtained all necessary terms of state-full temporal-difference learning with $\epsilon$-greedy strategies in value space $\varvec{q}$. Using an efficient python implementation, we can apply those learning equations for simulation studies to investigate multi-agent learning phenomena in a fast and deterministically reproducible way.

### Batch learning

The batch reinforcement learning problem was originally defined as learning the best strategy from a fixed set of a-priori-known transition samples^58^. However, our goal is to construct an algorithm able to interpolate between the fully online and fully deterministic version of the temporal-difference reinforcement learning process. The learning process is divided into two phases, an interaction phase, and an adaptation phase. During the interaction phase, the agent keeps its strategy fixed while interacting with its environment for *K* timesteps, collecting state, action, and reward information. During the adaptation phase, the agent uses the collected information to update its strategy. Key is the use of two state-action-value tables, one for acting ($q_\text {act}$), the other for improved value estimation ($q_\text {val}$). While $q_\text {act}$ is kept constant during the interaction phase, $q_\text {val}$ is iteratively updated^56,57^.

Furthermore, we use an auxiliary, time-dependent learning rate $\alpha (s, a, t_{s, a})$ for $q_\text {val}$ and a global learning rate $\alpha$ for $q_\text {act}$. Here $t_{s, a}$ is the local time of the state-action pair (*s*, *a*), which is given by the number of times the state-action value $q_\text {val}(s, a)$ has been updated during the batch. Since the environment is kept fixed for the duration of the batch, each sample in the batch should be valued equally. This can be achieved by using a state, action, and time-dependent learning rate $\alpha (s, a, t)=\frac{1}{t+1}$ (Algorithm 1).
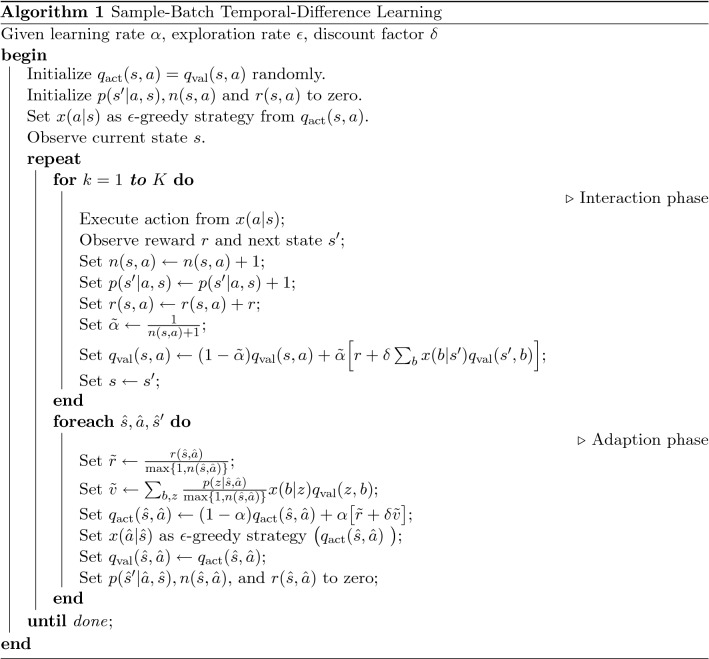


## Supplementary Information

Supplementary Information.

## Data Availability

Code to reproduce all results is available at: https://github.com/wbarfuss/intrinsic-fluctuations-cooperation and is archived at: https://doi.org/10.5281/zenodo.7303593.
