# Supplementary material for: Intrinsic fluctuations of reinforcement learning promote cooperation
Source: Sci Rep. 2023 Jan 24;13:1309. doi: 10.1038/s41598-023-27672-7 (PMC9873645; doi:10.1038/s41598-023-27672-7)
Supplement: Supplementary file 1 — Supplementary Information. [file 41598_2023_27672_MOESM1_ESM.pdf]

Supplementary Information

Here we provide the supplementary information for the manuscript: "Intrinsic fluctuations of reinforcement learning promote cooperation". In Figure SI 1, we plot the results of our robustness analysis for the environmental parameters $T = 1.5$ and $S = -0.2$, and in Figure SI 2 we plot the results of our robustness analysis for the environmental parameters $T = 1.25$ and $S = -0.25$. We see that levels of cooperation close to one are possible for both environments. These can be achieved on relatively short time scales compared to the timescale required by the algorithm without batches.

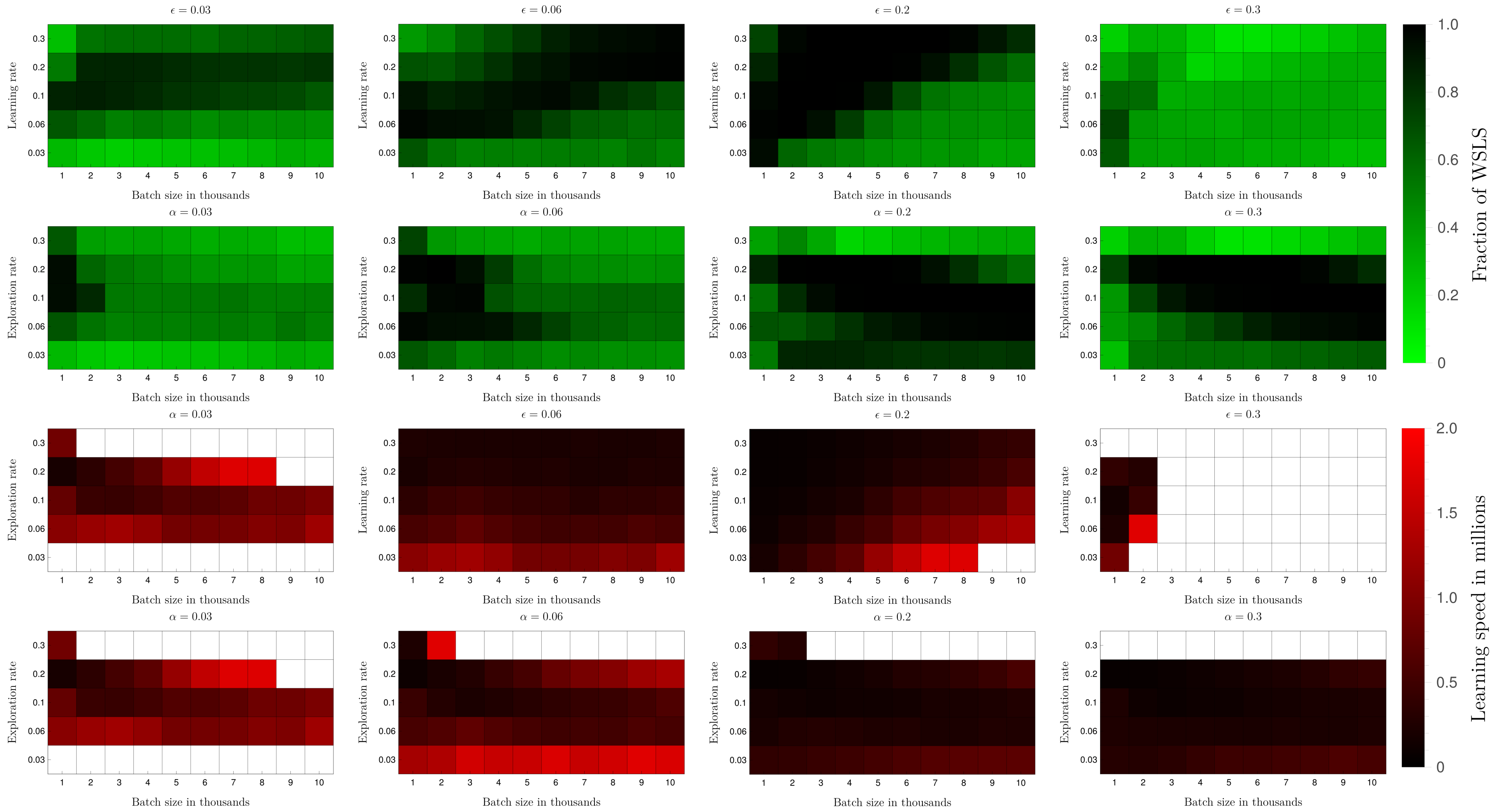

Figure SI 1: Plots showing the robustness of the results for different parameter values. The top two rows show the fraction of trajectories (1000 samples) that end in the WSLS strategy pair at time 2×10^6. The bottom two rows show the time it takes for the fraction of trajectories in the WSLS strategy pair to reach 0.4 in millions of time steps (we use white to represent trajectories that never reached 0.4). The x-axis always represents the batch size in thousands, and the y-axis represents either the learning rate α or the exploration rate ϵ. In all cases, we set $\delta = 0.99$.

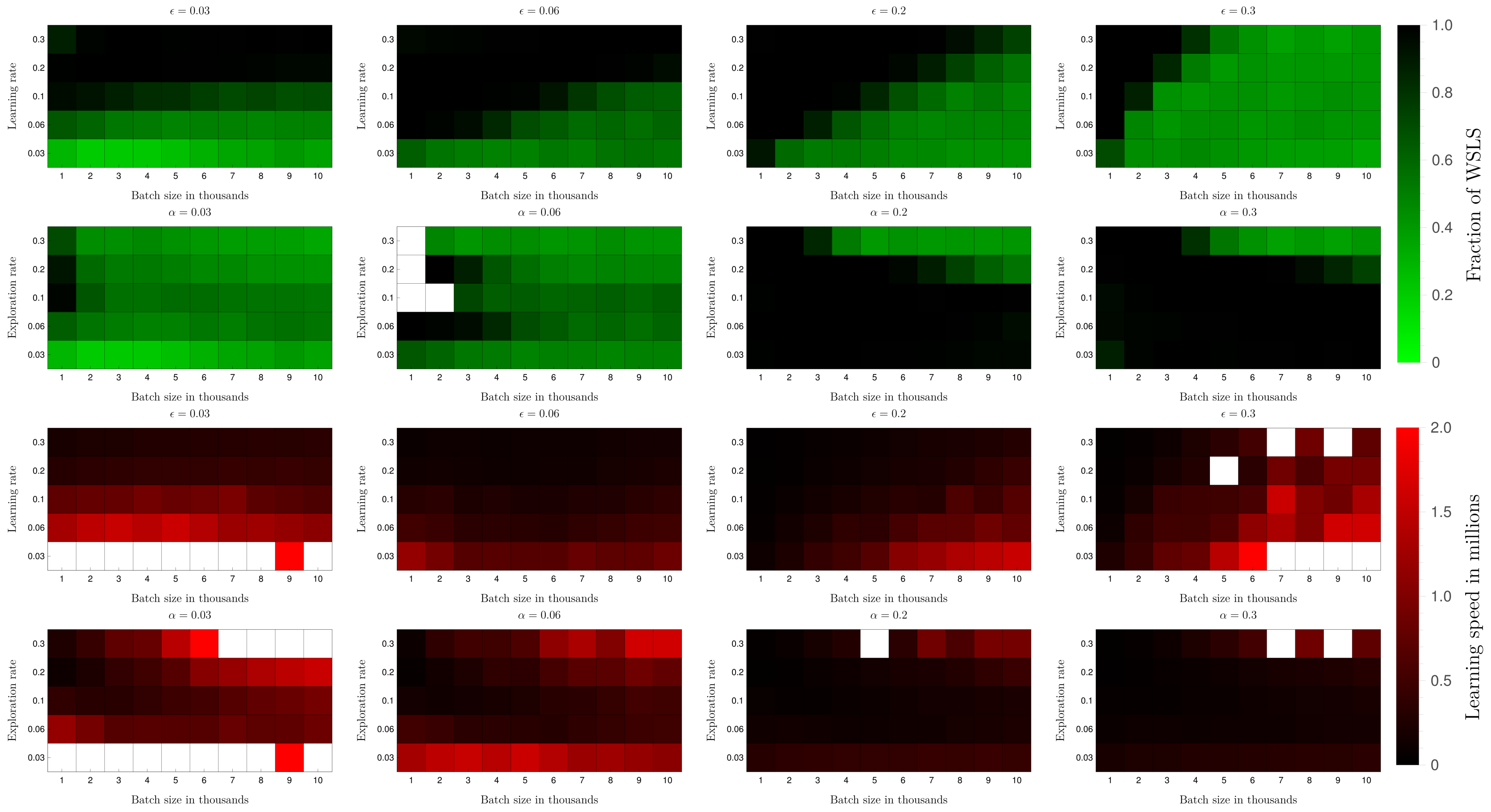

Figure SI 2: Plots showing the robustness of the results for different parameter values. The top two rows show the fraction of trajectories (1000 samples) that end in the WSLS strategy pair at time 2×10^6. The bottom two rows show the time it takes for the fraction of trajectories in the WSLS strategy pair to reach 0.4 in millions of time steps (we use white to represent trajectories that never reached 0.4). The x-axis always represents the batch size in thousands, and the y-axis represents either the learning rate α or the exploration rate ϵ. All plots are for the environment with $T = 1.25$ and $S = -0.25$. In all cases, we set $\delta = 0.99$.
